# Application of Infrared Thermography in Identifying Plant Oils

**DOI:** 10.3390/foods13244090

**Published:** 2024-12-17

**Authors:** Maria Marudova, Sotir Sotirov, Nadezhda Kafadarova, Ginka Antova

**Affiliations:** 1 Faculty of Physics and Technology, Plovdiv University “Paisii Hilendarski”, 4000 Plovdiv, Bulgaria; sotir@uni-plovdiv.bg (S.S.); nadezhda.kafadarova@uni-plovdiv.bg (N.K.); 2 Faculty of Chemistry, Plovdiv University “Paisii Hilendarski”, 4000 Plovdiv, Bulgaria; ginant@uni-plovdiv.bg

**Keywords:** thermography, vegetable oils, identification

## Abstract

In this article, we present a unique system for identifying edible oils through the analysis of their thermophysical properties. The method is based on the use of active infrared thermography. The heating of the oils results from the optical absorption of laser radiation at a specified wavelength. This approach enables greater selectivity in differentiating between various types of edible oils, as the results depend not only on the thermal properties of the specific oils but also on their optical properties, which are uniquely characteristic of each oil. Additionally, the developed system provides a detailed visualization of spatial temperature gradients within the sample’s volume, as well as their changes over time. It overcomes the limitations of other methods that determine only the thermal conductivity coefficients of oils through resistive heating of the sample. In this article, four types of vegetable oils (extra virgin olive oil, sesame oil, sunflower oil, and rapeseed oil) have been studied. Fatty acid analysis, differential scanning calorimetry, and UV-VIS spectroscopy have been used to determine the authenticity, moisture content, and optical properties of the studied samples. The developed system allows for the visualization and determination of the emerging temperature gradients in the sample volume.

## 1. Introduction

The increasing prevalence of adulteration, mislabeling, and food fraud has made the authentication of vegetable oils a pressing issue. With growing consumer demand for vegetable oils driven by evolving dietary habits and heightened health awareness, the industry has become a target for unethical practices [1]. These actions introduce substandard or counterfeit products into the supply chain, undermining quality and trust. To address this challenge, there is an urgent need to develop and implement robust methods for ensuring the quality and certification of vegetable oils. There are a variety of instrumental methods for analyzing vegetable oils, such as chromatography, nuclear magnetic resonance (NMR), spectroscopy, differential scanning calorimetry (DSC), biosensors (electronic nose and electronic tongue), etc. [2]. However, several major shortcomings of these methods can be identified. Chromatographic methods (GC, HPLC, HPTLC) are accurate but expensive and time-consuming. They require large quantities of solvents that are not environmentally friendly, as well as experienced staff to operate the apparatus. NMR and Raman spectroscopy (RS) require expensive instruments and trained personnel. Some methods like spectroscopy and DSC show low sensitivity. Biosensors and dielectric spectroscopy are highly dependent on environmental factors such as humidity and temperature.

These are widely used methods for the identification of oils, related to their thermal properties. Over the years, different techniques have been developed for the experimental investigation of the thermal conductivity properties of edible oils [3]. To accurately determine thermal conductivity, multiple specific parameters must be considered, such as molecular weight (related to the mobility and kinetic energy of molecules), the acentric factor (characterizing the shape of the molecules), enthalpy, and the compressibility coefficient. The temperature dependence of the viscosity of a particular oil also significantly affects its thermal conductivity. On a microscopic level, this can be explained by the increased mobility of molecules at higher temperatures, which reduces the efficiency of heat transfer. Consequently, the viscosity and, to a lesser extent, the thermal conductivity of oils decrease with increasing temperature.

Another factor influencing thermal conductivity is the presence of unsaturated fatty acids. Oils with a higher content of unsaturated fatty acids have greater thermal conductivity [4]. Refined vegetable oils are less susceptible to a decrease in thermal conductivity with increasing temperature.

In most studies, the composition of the measured samples of vegetable oils is not fully specified, leading to discrepancies in the thermal conductivity data reported in the literature. These variations are typically attributed to differences in the composition and structure of the same oil. Another factor contributing to the discrepancies may be the measurement method and the conditions under which the experiment is conducted. For example, steady-state methods take more time than transient hot-wire (THW) methods, which may cause changes in the samples during measurement, such as moisture loss. Thermal conductivity is highly dependent on moisture content, temperature, and the material’s structure.

The most commonly used methods include steady-state techniques, the temperature oscillation method (also known as the modified Ångström method) [5], the transient hot-wire (THW) method using resistive or thermocouple-based temperature measurements [6], the flash method, as well as acoustic, photothermal, and light-scattering-based methods [7,8,9]. The accurate measurement of thermal conductivity presents a significant challenge, with the main difficulties involving the prevention of heat transfer through convection in the liquid and controlling the temperature gradients that arise during measurement. These challenges can be successfully addressed by employing thermographic techniques to monitor temperature changes in the sample, enabling precise temperature measurements across the entire surface of the investigated sample.

The method of infrared thermography is based on analyzing the infrared radiation emitted by an object. As is well known, the intensity of this radiation is directly related to the object’s temperature.

The measurement results are presented as a color map, where each color corresponds to a specific temperature. This provides a clear representation of the two-dimensional spatial distribution of surface temperature for the object under investigation. These maps are displayed as digital images known as thermograms.

This method enables a non-contact temperature measurement across the entire surface of the object, which is a significant advantage over other commonly used methods, such as those involving thermocouples, thermoresistors, and similar devices. It overcomes the key limitations of these methods, such as measuring temperature at only a single point on the surface, which does not provide a clear understanding of the temperature distribution across different areas.

Another major drawback addressed by thermography is the need to establish good thermal contact, often requiring the use of thermal interface materials. Additionally, many cases necessitate the development of specialized fixtures to attach the thermal sensor to the surface of the investigated object, introducing additional sources of error in the temperature measurement.

For an accurate temperature measurement, an infrared camera does not merely rely solely on the object’s temperature but also considers its emissivity. Additionally, the object may reflect radiation from the surrounding environment. Another significant factor affecting the thermographic measurement results is atmospheric absorption, which depends on the humidity of the environment and the distance between the object and the camera.

To compensate for these errors, the infrared camera automatically applies the necessary corrections in real time. However, the following object parameters must be specified beforehand: the object’s emissivity, ambient temperature, the distance between the object and the camera, and relative humidity.

These properties make thermography a preferred method for temperature measurement in a wide range of applications, including industry, medicine, agriculture, and more. In recent years, thermography has begun to be applied in various areas of the food industry, particularly for assessing food quality [10,11,12].

Infrared thermography studies for characterizing vegetable oils can include various applications based on the ability of infrared cameras to detect temperature differences and the thermal behavior of oils. For example, Ref. [13] presents research results on detecting adulteration in unprocessed extra virgin olive oils (EVOOs) using thermography combined with deep learning. Therefore, the use of infrared thermography in the analysis of vegetable oils provides valuable insights into their thermal behavior, quality, and authenticity while also being a non-invasive and rapid technique.

In this article, we present a unique system for identifying edible oils through the analysis of their thermophysical properties. This method is based on the use of active infrared thermography. The heating of the oils results from the optical absorption of laser radiation at a specified wavelength.

This approach enables greater selectivity in differentiating between various types of edible oils, as the results depend not only on the thermal properties of the specific oils, as with the aforementioned measurement methods, but also on their optical properties, which are uniquely characteristic of each oil.

Additionally, this method provides a detailed visualization of spatial temperature gradients within the sample’s volume, as well as their changes over time. It overcomes the limitations of other methods that determine only the thermal conductivity coefficients of fats through the resistive heating of the sample.

## 2. Materials and Methods

### 2.1. Materials

Refined sunflower oil (SFO), extra virgin olive oil (EVOO), rapeseed oil (RSO), and sesame oil (SO) were bought from a Bulgarian local market. All of them were packaged in original unopened dark glass bottles and stored in a refrigerator at a temperature of 5 °C. Before the analyses, the oils were tempered at a temperature of 20 °C for 1 h. All other chemicals used in the physical and chemical testing were of analytical grade.

### 2.2. Determination of Fatty Acid Composition

The fatty acid composition of the oils was determined using the method of gas chromatography (GC), according to the ISO 12966-4:2015 standard [14]. Fatty acid methyl esters (FAMEs) were prepared by pre-esterification of the samples with 2% sulfuric acid in absolute methanol at 50 °C based on the ISO 12966-2:2017 standard [15]. Determination of FAMEs was performed on Agilent 8860 gas chromatograph, which was equipped with a capillary column DB Fast FAME (30 m × 0.25 mm × 0.25 μm film thickness) and a flame ionization detector. The column temperature varied from 70 °C (1 min) to 180 °C, at a rate of 6 °C/min, and then from 180° to 250 °C at a rate of 5 °C/min. The injector temperature was 270 °C, and that of the detector was 300 °C. Nitrogen was used as a carrier gas. Identification was carried out by comparison of the retention times of a standard mixture of FAME.

### 2.3. Differential Scanning Calorimetry

The crystallization and melting phenomena of the investigated oils were examined by the method of differential scanning calorimetry. DSC 204F1 Phoenix produced by Netzsch Gerätebau GmbH, Selb, Germany, was used in these measurements. The instrument was preliminary calibrated for heat flow and temperature by the indium standard (T_m_ = 156.6 °C, ΔH_m_ = 28.5 J/g). A total of 10 mg of the oil samples was placed in aluminum pans and hermetically sealed. An identical empty pan was used as a reference. The following temperature protocol was followed: cooling down from 25 °C to −70 °C with a cooling rate of 2 °C/min; isothermal step at −70 °C for 10 min; and heating from −70 °C up to 100 °C with a heating rate of 5 °C/min. The experimental data were evaluated using Netzsch Proteus—Thermal Analysis software (Version 6.1.0B, Selb, Germany).

### 2.4. UV-VIS Spectrophotometry

The UV-VIS analysis was performed on the Metertech UV/VIS Spectrophotometer SP-8001 (Metertech Inc., Taipei, Taiwan) in the wave range from 190 nm to 800 nm. Samples were measured without dilution.

### 2.5. Thermographic System for Vegetable Oils’ Identification

**Figure 1:**
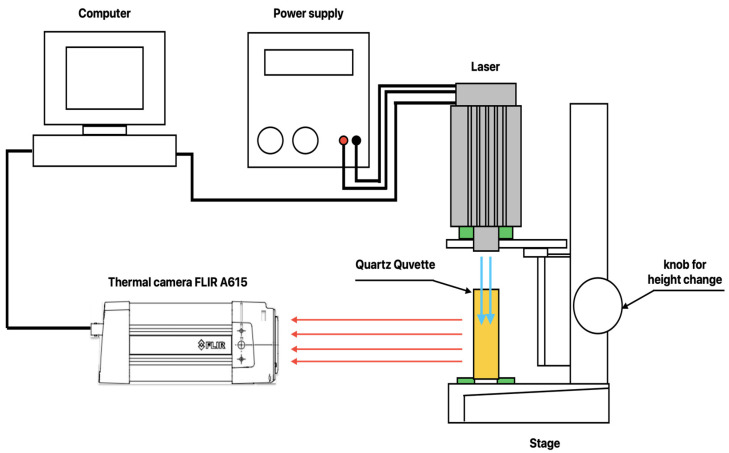
The block diagram of the system for vegetable oils’ identification.

The block diagram of the developed unique system for vegetable oils’ identification is shown in Figure 1.

The samples of different types of oils under investigation are placed in a quartz cuvette with a height of 45 mm, a width of 12.5 mm, and a wall thickness of 2.5 mm. The cuvette is positioned at the center of the base of a laboratory stand using a holder made from polylactic acid. The holder contacts the bottom of the cuvette at four points, each with an area of 1 mm^2^, thus limiting the heat transfer between the cuvette and the base of the stand. Above the cuvette, a laser source is mounted in such a way that the axis of the laser beam aligns with the center of the cross-section of the cuvette. This design allows for the adjustment of the laser height relative to the base of the stand. The laser source used is the LaserTree LT-20W-A from Shenzhen Guangchuangfeng Technology Co., Ltd., Shenzhen, China, with a power of 4W, a working wavelength of 450 nm ± 10 nm, and a focal length of 35 mm. To accurately determine the central wavelength of the laser used at the ambient temperature at which the experiments were conducted, a HAMAMATSU C12666MA spectrometer was used. The stability of the laser’s emitted optical power was investigated using a ThorLabs PM100 power meter operating in the spectral range of 400 nm–1100 nm. The obtained results show that, with an electrical power supply of 12 V, 1.81 A, and a controlled ambient temperature of 22 °C, the average output power of the laser in continuous operation mode over 1 h is 3.92 W ± 0.09 W.

The power supply is provided by a laboratory power supply unit Teledyne T3PS13206, Teledyne, China, with power regulation achieved through pulse-width modulation.

The absorption of the laser radiation by the sample material leads to its heating, which results in the generation of infrared (IR) radiation. The intensity of the infrared light emitted by the sample is proportional to its temperature. To determine the spatial distribution of the temperature field within the sample, a FLIR A615 thermocamera from Teledyne FLIR LLC, Sweden, is used. The camera is positioned 30 cm away from the sample and is aligned so that the wall of the cuvette being imaged is perpendicular to the optical axis of the lens, f = 24.6 mm. To reduce errors due to reflection, the matte surface of the cuvette wall is imaged. The camera is connected to a personal computer, which records and visualizes the obtained images.

All components of the system are placed in a laboratory with controlled humidity and temperature, without windows. The minimum distance between the measurement system and the walls of the laboratory is 1.5 m. On the other hand, the laboratory room is completely darkened to eliminate the influence of other sources of infrared radiation on the measurement.

Immediately before the measurement, 4 mL of the sample material is placed in the cuvette and tempered for a specified period of time in the laboratory room. This process is recorded with the thermographic camera until a uniform temperature distribution within the sample (within 0.5 °C) is achieved, and its temperature becomes close to that of the surrounding environment. For each of the examined oils, 5 samples are taken, and consecutive measurements are conducted. The values from the five measurements were averaged.

**Figure 2:**
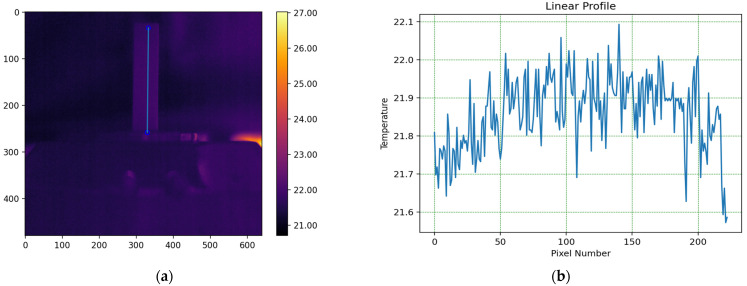
Thermogram taken after the completion of the sample tempering process. (**a**) Thermogram of the sample before the measurement process; (**b**) temperature distribution profile along the longitudinal axis of the cuvette, shown as the blue line in (**a**).

Figure 2 shows a thermogram taken after the tempering process of the sample is completed.

The blue line in Figure 2a represents the position of the temperature profile shown in Figure 2b.

## 3. Results and Discussion

### 3.1. Fatty Acid Composition of the Investigated Oils

Fourteen fatty acids were identified in the composition of SFO, SO, EVOO, and RSO. The fatty acids with the highest content are presented in Table 1. The amount of the other fatty acids for all samples was detected in quantities lower than 0.5%.

The major component in sunflower oil was found to be linoleic acid (54.0%), followed by oleic acid (34.5%). The fraction of saturated fatty acids was represented by palmitic acid (6.4%) and stearic acid (3.3%). According to another report of Garcés et al. [16], the content of linoleic acid in sunflower oil was found to be higher (58.0%) than that in the current research, while the content of oleic acid was lower (29.0%).

The fatty acid composition of the glyceride oils can vary within certain limits, depending on the species, the variety, the geographical origin, the climatic conditions, and the method of cultivation [17].

**Table 1:** Fatty acid composition of the investigated oil.

Fatty Acid Composition, %		SFO	SO	EVOO	RSO
C_16:0_	Palmitic	6.4	10.4	13.2	4.4
C_18:0_	Stearic	3.3	2.7	1.4	1.4
C_18:1_	Oleic	34.5	36.7	72.9	64.7
C_18:2_	Linoleic	54.0	48.3	9.0	18.8
C_18:3 (n-3)_	α-Linolenic acid	-	-	-	8.5
Saturated fatty acids, %		11.0	13.8	15.5	6.4
Unsaturated fatty acids, %		89.0	86.2	84.5	93.6
Monounsaturated fatty acids, %		34.9	37.1	74.5	66.2
Polyunsaturated fatty acids, %		54.1	49.1	10.0	27.4

Similar results were observed for sesame oil, with the linoleic acid content at 48.3% and oleic acid at 36.7%. The levels of saturated fatty acids were slightly higher (10.4% palmitic acid and 2.7% stearic acid, respectively) compared to those found in sunflower oil. The data about sesame oil according Shotorbani et al. [18] correspond with our results.

The major component of extra virgin olive oil was oleic acid (72.9%), followed by palmitic acid (13.2%), linoleic acid (9.0%), and stearic acid (1.4%). The found composition corresponds to the standard developed by the International Olive Council [19].

Similarly to EVOO, RSO was again high in oleic oil, with an oleic acid content of 64.7% and linoleic acid content of 18.8%. It also contained a higher amount of α-linolenic acid—8.5%. Its saturated fatty acid content is the lowest among the examined samples. The obtained results correspond to those reported by Banaś at al. [20], based on which rapeseed oil contains between 6% and 14% α-linolenic acid, 50–60% oleic acid, and <7% saturated fatty acids.

Monounsaturated fatty acids (MUFAs) and polyunsaturated fatty acids (PUFAs) constitute a fraction of unsaturated fatty acids. Unsaturated fatty acids predominated in all oils. The amount of saturated fatty acids (SFAs) was found to be significantly lower.

The established fatty acid composition of the studied oils and its correspondence to the data found in the literature give us reason to consider the authenticity of the samples we examined.

### 3.2. Thermal Behavior of the Oils

**Figure 3:**
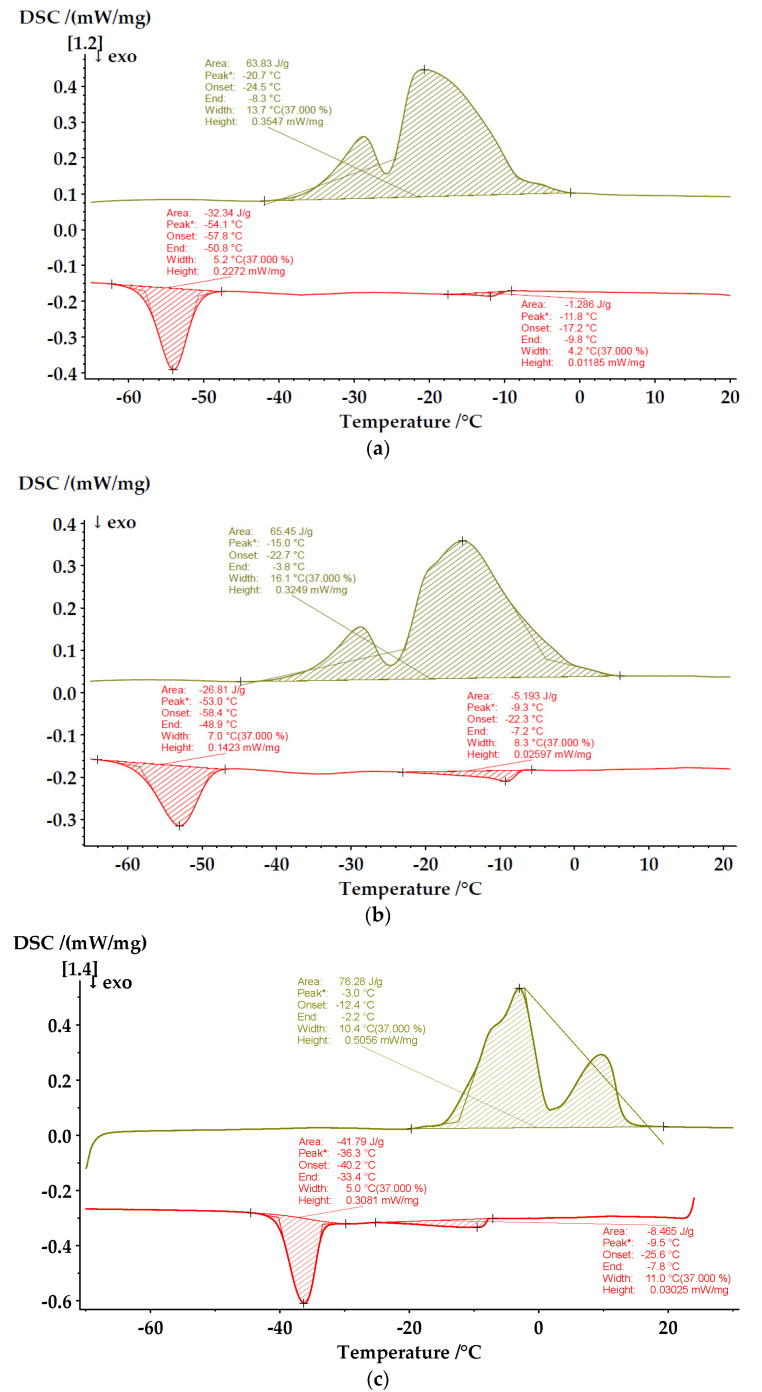

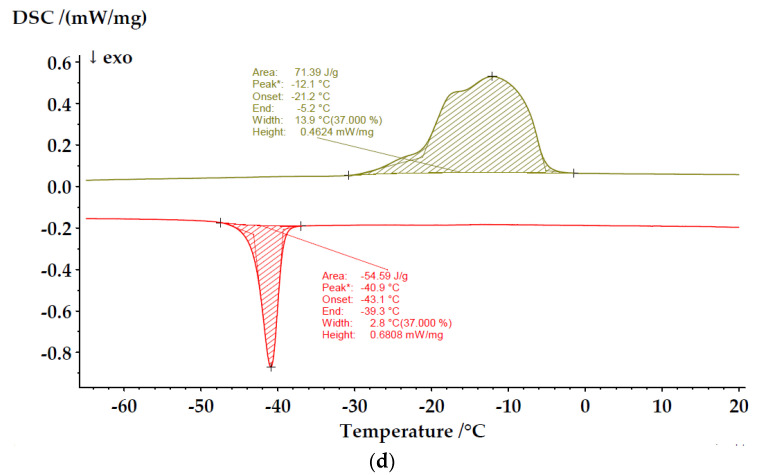
Crystallization and melting transitions of (**a**) SFO, (**b**) SO, (**c**) EVOO, and (**d**) RSO.

In order to confirm the authenticity of the samples and establish moisture in them, a thermal analysis was carried out using the method of differential scanning calorimetry. The thermal transitions in the investigated oils are presented in Figure 3a–d. It can be seen that the DSC profiles differ significantly from each other due to the differences in the fatty acid composition of the oils.

The cooling DSC curve of SFO (Figure 3a) presents two exothermic peaks associated with two distinct crystallization events. The first one appears at T_p_ = −11.8 °C and can be attributed to the phase transition of a small oil fraction containing mainly saturated FAs such as palmitic and stearic acid. The second exothermic peak has been shown at T_p_ = −54.1 °C corresponding to the phase transition of the low-melting highly unsaturated oil fraction. The heating DSC curve shows a double endothermic peak with a high temperature shoulder, corresponding to the melting phenomena.

The observed temperatures of the phase transitions are in accordance with those reported in the literature [21].

The DSC thermograms of sesame oil are very similar, with the larger area of the high-temperature crystallization transition accounting for the higher saturated fatty acid content.

The cooling curve for EVOO is characterized again with two endothermic peaks (Figure 3c). During heating, the increase in temperature caused the melting of the highly unsaturated fraction at −3 °C, followed by that of the more saturated ones (1) at about 10 °C. These results are in good agreement with those previously discussed [22].

In the cooling DSC curve of RSO, only one exothermic peak at −41 °C is detected. This is associated with the crystallization of unsaturated acids, which are about 94% of all fatty acids in the oil. Upon heating, a broad endothermic peak is observed between −30 °C and 0 °C. Similar DSK thermograms were observed by Islam et al. [23].

No phase transitions of water were registered in any of the oil samples, which gives us reason to consider that there is no moisture in the studied oils.

### 3.3. UV-VIS Absorption of the Oils

**Figure 4:**
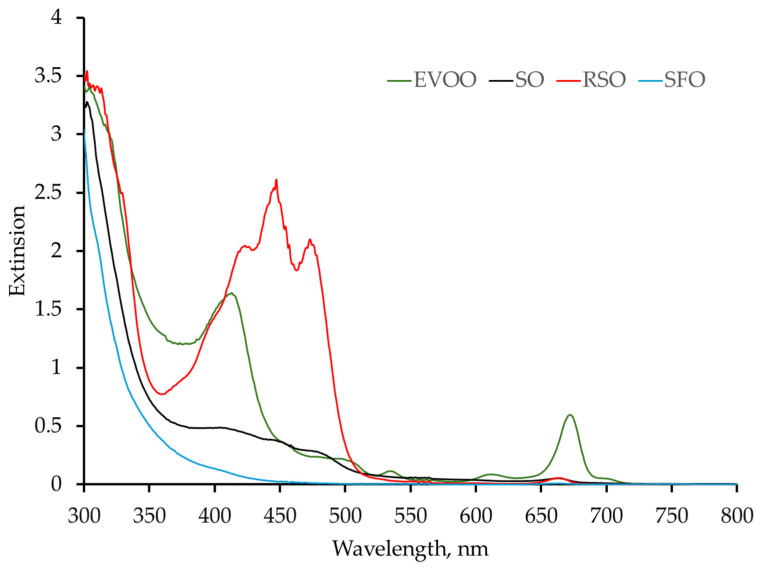
UV-VIS spectra of EVOO, SO, RSO, and SFO.

In the present study, the method of adsorption spectroscopy was used to determine the absorption bands of vegetable oils. Their UV-VIS spectra are presented in Figure 4. The absorption properties depend on the content of dye components such as chlorophyll, fovitin, carotenoids, and lutein.

The EVOO samples show high absorption values around 301 nm, 373 nm, 411 nm, and 672 nm. These bands are associated with the presence of polyphenols, carotenoids (400–450 nm), and chlorophyll (670 nm), as reported by other authors [1,24].

Absorption peaks of SO appear at presses sesame oil at 313 nm, 410 nm, and 662 nm, which are mainly due to the presence of polyphenols and chlorophyll [25]. The RSO spectra are characterized with three absorption bands of carotenoids in the wavelength range of 420–480 nm [26]. Sunflower oil practically does not absorb in the range from 430 nm to 800 nm.

The conducted UV-VIS spectral analysis does not aim to provide a detailed characterization or identification of the four types of oils under investigation. The primary objective of this analysis is to determine the absorption coefficients of the different oils at a wavelength of 450 nm, which corresponds to the operating wavelength of the semiconductor laser used in this study. This is essential since the heating of the oils is achieved optically through the absorption of laser energy. The absorption coefficient values are crucial for accurately interpreting the results obtained from the infrared thermography.

Based on the absorption spectra obtained, it can be concluded that the absorption of the oils at a wavelength of 450 nm, corresponding to the laser wavelength used in the research, is significantly different, being strongly manifested in RSO (2.41), followed by EVOO (0.37), SO (0.36), and sunflower oil (0.07). It is very close for EVOO and SO.

### 3.4. Study of Edible Oils Using the Developed Thermographic System for Vegetable Oils’ Identification

The measurement process for each sample lasts 160 s after the laser source is activated. The interaction of the laser radiation with the sample material leads to an increase in its temperature.

The laser startup is synchronized with the operation of the thermovision camera, which automatically captures the change in the sample’s temperature at 20 s intervals. This allows for the visualization of temperature gradient differences within the sample during the measurement.

**Figure 5:**
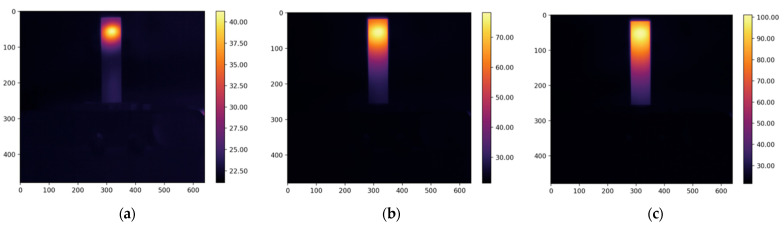
The obtained thermograms captured at (**a**) 20 s, (**b**) 80 s, and (**c**) 160 s from the study of extra virgin olive oil.

**Figure 6:**
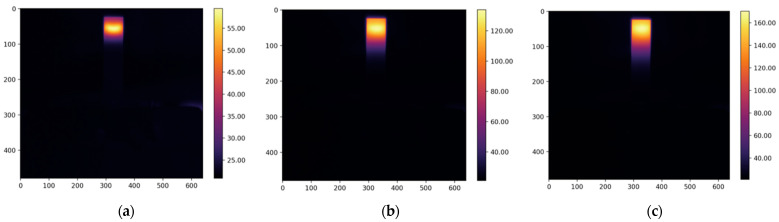
The obtained thermograms captured at (**a**) 20 s, (**b**) 80 s, and (**c**) 160 s from the study of rapeseed oil.

**Figure 7:**
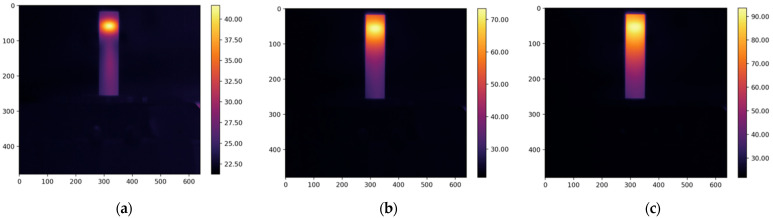
The obtained thermograms captured at (**a**) 20 s, (**b**) 80 s, and (**c**) 160 s from the study of sesame oil.

**Figure 8:**
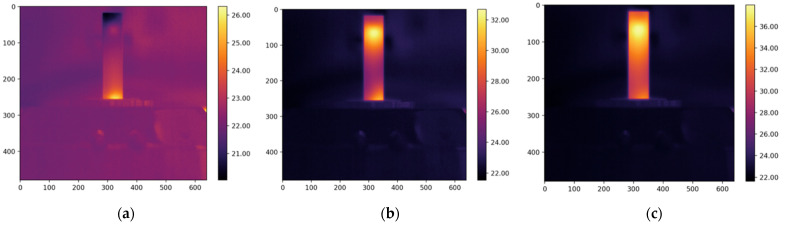
The obtained thermograms captured at (**a**) 20 s, (**b**) 80 s, and (**c**) 160 s from the study of sunflower oil.

The resulting thermograms, captured at 20, 80, and 160 s from the study of four types of oil, are presented in Figure 5, Figure 6, Figure 7 and Figure 8.

The thermograms clearly show the differences in temperature distribution among the different samples, which are due to differences in both the absorption coefficients at a wavelength of 450 nm and the varying thermal conductivity coefficients of the different oils. For each thermogram, a temperature profile has been created, presenting information on the temperature values along the longitudinal axis of the studied sample, which coincides with the axis of the laser beam, as shown in Figure 2.

**Figure 9:**
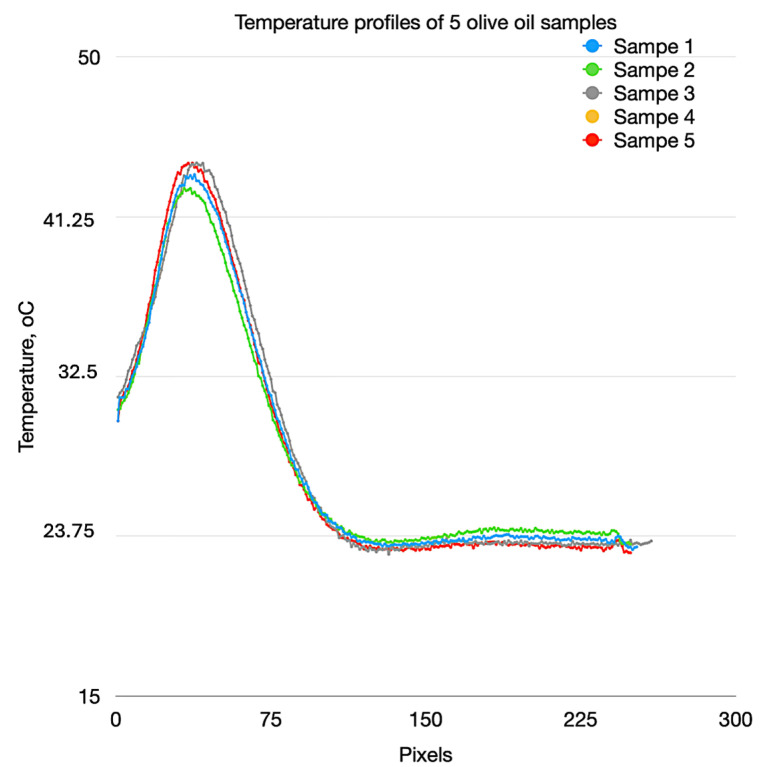
Measured temperature profiles of 5 samples of olive oil at the 20th s after the start of the analysis.

To determine the operational stability of the system, experiments have been conducted in which a series of samples of the same oil have been measured. After each measurement, the content of the analyzed oil in the cuvette has been replaced with a fresh sample. For example, in Figure 9, the temperature profiles of olive oil from five measurements at the 20th s after the start of the analysis are presented. The measurements were performed at 1 h intervals. The average value of the maximum temperature at the top of the peak is (41.28 ± 1.68) °C.

**Figure 10:**
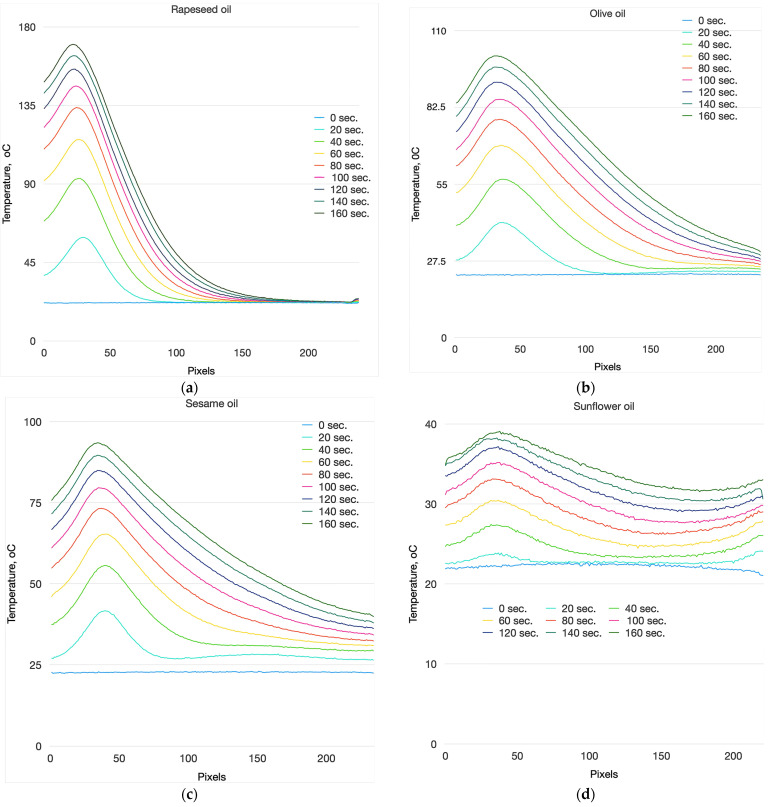
Temperature profiles of (**a**) rapeseed oil; (**b**) EVOO; (**c**) sesame oil; (**d**) sunflower oil.

Figure 10 shows the average temperature profiles of the four types of oils studied. It clearly shows the differences in the measured temperature profiles of the four types of oils. The distance between two adjacent pixels is 0.15 mm. As can be seen, the highest temperature measured in the sample is approximately 4.6 mm from the top of the cuvette, which corresponds to the focal zone of the laser beam. In this zone, the energy density of the laser radiation is the highest, which also leads to the highest temperature. As the distance along the longitudinal axis of the cuvette increases from the focal zone towards the bottom, a decrease in temperature is observed.

From the graphs presented, it is evident that the temperature gradient is most pronounced in rapeseed oil, where the highest maximum temperatures are achieved. Additionally, at a distance of 28.35 mm from the top of the cuvette, the temperature profiles converge into a single line.

The maximum temperatures in the profiles of the investigated oils are presented in Table 2. The maximum temperatures in the profiles of sesame oil and olive oil are approximately the same up to 40 s, but despite this, a significant difference in the temperature distribution along the longitudinal axis of the sample is noticeable, with sesame oil being significantly more uniform. The most noticeable difference in the temperature profile is observed in sunflower oil, where the temperature gradient along the length of the sample is the least pronounced.

The occurrence of a temperature gradient in the sample is mainly due to the process of the absorption of the laser radiation by the substance, as well as to the processes related to heat transfer in the volume of the sample. These processes are related to the physico-chemical and thermo-physical properties of the specific substance and can therefore be used as criteria for determining the different types of oils. Also, the conducted measurement makes it possible to determine the change in the maximum temperature of the sample over time.

**Figure 11:**
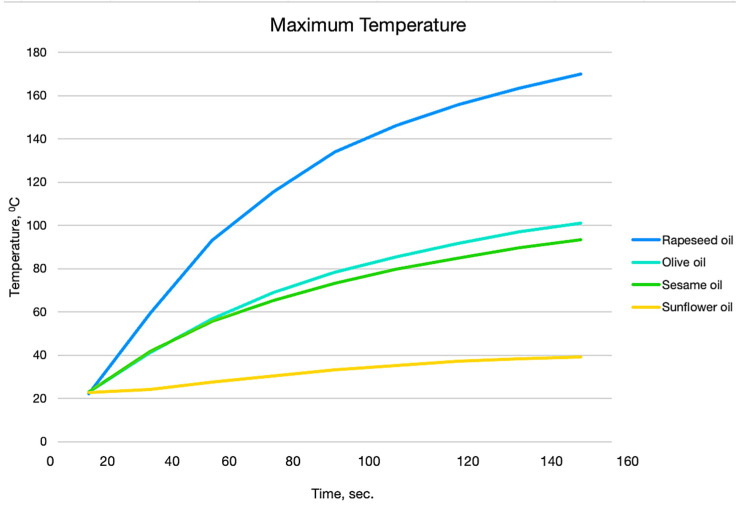
Graphical dependences of the variation in the maximum temperatures for each sample depending on the time.

Figure 11 presents graphical dependences of the variation in maximum temperatures for each sample depending on time.

The lowest temperature change is observed for sunflower oil, which is due to the low absorption coefficient at the wavelength of the laser source used (450 nm). For sesame oil and olive oil, the temperature change curves overlap up to 40 s. This has required studies to be conducted over a longer period of time, where temperature differences become significant and allow these oils to be identified. The maximum temperature increases fastest with respect to time in the case of rapeseed oil, as at 160 s it reaches 169.96 °C.

**Figure 12:**
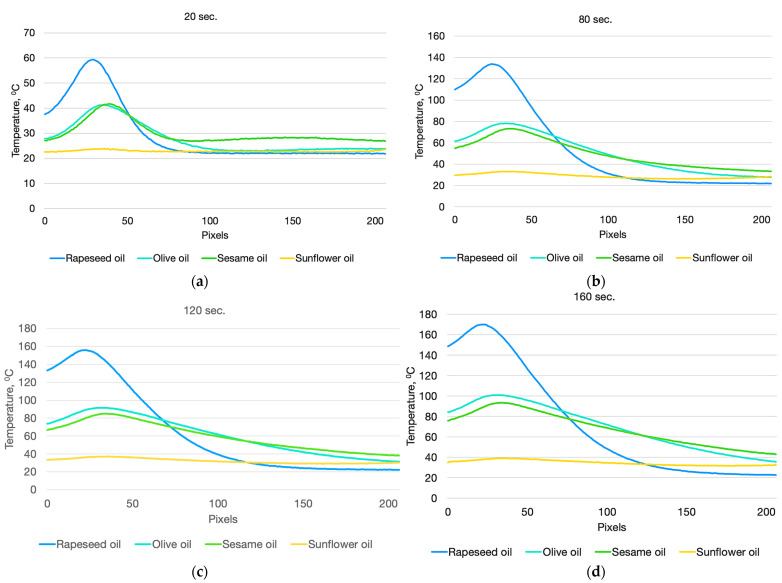
Change in the temperature profiles of the studied oils over time: (**a**) at 20 s from the measurement; (**b**) at 80 s from the measurement; (**c**) at 120 s from the measurement; (**d**) and at 160 s from the measurement.

Figure 12 shows the graphs of the temperature profiles for each of the studied oils at different exposure times to the laser radiation. The developed measurement method used in the experiments, based on infrared thermography, allows for the observation and examination of the temperature gradients that arise in the studied volume, as well as the changes in these gradients over time. As seen from the figure, the temperature profiles of the studied oils differ significantly, which provides a strong basis for identifying the thermal behavior, quality, and authenticity of various edible oils using infrared thermography.

The temperature profiles presented in Figure 11 allow us to clearly distinguish the different types of oils by means of the temperature gradient. The largest temperature gradient is that of rapeseed oil, which is determined by the large absorption coefficient, due to which a large part of the laser energy is absorbed in a small volume of the sample and a sharp increase in temperature is created. A sharp drop in temperature is observed up to 110 pixels, after which the temperature changes within small limits.

The most uniform temperature distribution is observed in the volume of sunflower oil. This is due to the extremely low absorption coefficient of laser energy in the volume of the substance which allows the laser beam to pass through the entire volume of the probe.

In sesame oil and olive oil, due to the close absorption coefficients, the same maximum temperature is observed in 20 s from the start of the experiment. However, the temperature distribution in the volume of the two oils differs significantly, which is due to the temperature dependence of the thermal conductivity coefficient. The steepness of the temperature profile of olive oil is greater than that of sesame oil. This is a definitive indication by which the two oils can be distinguished.

**Figure 13:**
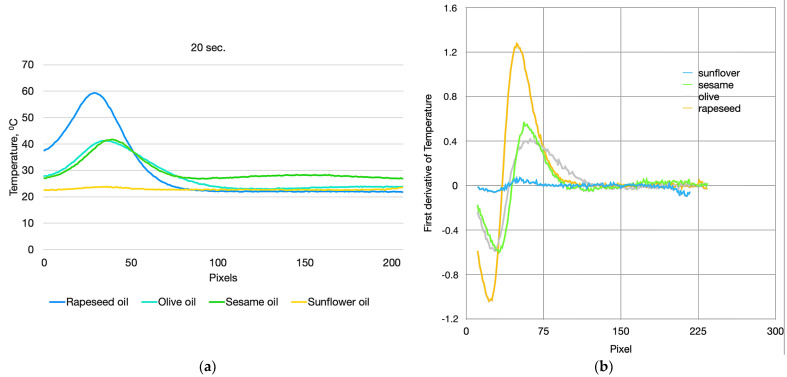
(**a**) Temperature profiles of the studied oils at 20 s from the measurement; (**b**) first derivative of temperature profiles of the studied oils at 20 s from the measurement.

In addition to the visual interpretation of the obtained temperature profiles, a quantitative analysis has been performed. To determine the magnitude of the temperature gradients for each temperature profile, the first derivative of temperature with respect to the camera pixels has been calculated. In the experimental setup configuration used, the vertical spatial resolution is 0.15 mm. As an example, Figure 13a presents the temperature profiles of the studied oils at 20 s from the measurement, while Figure 13b shows the corresponding graphs of the first derivatives for each temperature profile. The highest temperature gradient is observed for rapeseed oil, where the maximum value of the first derivative of temperature in the steepest part of the temperature peak is 1.21 °C/0.15 mm. Although the peak maximum temperatures in the temperature profiles of olive oil and sesame oil are approximately the same (Figure 13a), the maximum steepness of their temperature peaks, determined by their first derivatives, differs. For olive oil, it is 0.4 °C/0.15 mm, while for sesame oil, it is 0.5 °C/0.15 mm.

The method we propose allows for different types of oils to be distinguished not only through visual analysis of their temperature profiles but also through quantitative determination of the magnitude of the temperature gradients

## 4. Conclusions

In this article, four types of vegetable oils (extra virgin olive oil, sesame oil, sunflower oil, and rapeseed oil) purchased from a Bulgarian local market have been studied.

A fatty acid analysis has been conducted, and the established fatty acid composition of the studied oils and its correspondence with the data from the literature confirm the authenticity of the studied samples.

The analyses conducted using differential scanning calorimetry indicate that no phase transitions of water have been registered in any of the samples of the studied oils. This provides grounds to conclude that the studied oils lack moisture. This is particularly important for conducting research and identifying the oils using the developed thermographic system. The presence of moisture is a source of error, as it has a significant impact on the thermal conductivity of the oils.

The absorption spectra have been examined using UV-VIS spectroscopy for each of the oils to determine their absorption coefficients at a wavelength 450 nm used to irradiate the probe.

The developed unique thermographic system for vegetable oils’ identification is based on a combination of the optical and thermal properties of oils. It analyses the shape of the temperature profiles in space and time, which are strictly specific for the studied oils. The system allows for the visualization and determination of the emerging temperature gradients in the sample volume.

The presented system is inexpensive and can be further optimized. The experiments have to be conducted in a thermographic laboratory, at controlled temperature and relative humidity of the environment. The measurement time is within 3 min and is negligible compared to that of traditional methods such as chromatography. The resulting temperature error is complex and depends on the stability of the laser radiation power and the temperature error of the thermal imaging camera.

Current research will be expanded in the direction of identifying the geographical origin of vegetable fats, establishing the degree of oxidation and detecting counterfeits.

## Figures and Tables

**Table 2:** Changes in the maximum temperature of the four types of oils over time.

Time, s	Olive Oil, °C	Rapeseed Oil, °C	Sesame Oil, °C	Sunflower Oil, °C
0	22.88 ± 1.62 ^b^	21 ± 0.75 ^a^	23.28 ± 1.25 ^b^	22.65 ± 0.48 ^b^
20	41.28 ± 1.68 ^b^	58.27 ± 1.41 ^c^	41.35 ± 1.57 ^b^	23.77 ± 0.69 ^a^
40	57.07 ± 1.68 ^b^	92.65 ± 1.53 ^c^	56.27 ± 1.16 ^b^	27.94 ± 0.38 ^a^
60	68.88 ± 1.92 ^c^	114.69 ± 2.58 ^d^	65.54 ± 1.56 ^b^	30.78 ± 0.79 ^a^
80	77.68 ± 1.21 ^c^	131.84 ± 1.53 ^d^	73.97 ± 0.55 ^b^	33.21 ± 0.68 ^a^
100	85.58 ± 0.61 ^c^	145.46 ± 2.48 ^d^	79.44 ± 1.41 ^b^	35.56 ± 0.5 ^a^
120	92.14 ± 1.48 ^c^	156.03 ± 2.32 ^d^	85.83 ± 0.84 ^b^	37.56 ± 0.71 ^a^
140	96.32 ± 1.32 ^c^	164.06 ± 1.94 ^d^	88.79 ± 1.38 ^b^	38.49 ± 0.36 ^a^
160	101.3 ± 1.15 ^c^	171.14 ± 1.62 ^d^	94.2 ± 0.92 ^b^	39.45 ± 0.52 ^a^

^a–d^: the different letters mean a significant difference between the average values of the maximum temperatures of the oils (horizontal rows), determined at the same heating time (*p* < 0.05).

## Data Availability

The original contributions presented in the study are included in the article, further inquiries can be directed to the corresponding author.
